# Provisioning of Game Meat to Rural Communities as a Benefit of Sport Hunting in Zambia

**DOI:** 10.1371/journal.pone.0117237

**Published:** 2015-02-18

**Authors:** Paula A. White, Jerrold L. Belant

**Affiliations:** 1 Center for Tropical Research, Institute of the Environment and Sustainability, University of California Los Angeles, Los Angeles, California, United States of America; 2 Carnivore Ecology Laboratory, Forest and Wildlife Research Center, Mississippi State University, Starkville, Mississippi, United States of America; University of Vermont, UNITED STATES

## Abstract

Sport hunting has reportedly multiple benefits to economies and local communities; however, few of these benefits have been quantified. As part of their lease agreements with the Zambia Wildlife Authority, sport hunting operators in Zambia are required to provide annually to local communities free of charge i.e., provision a percentage of the meat obtained through sport hunting. We characterized provisioning of game meat to rural communities by the sport hunting industry in Zambia for three game management areas (GMAs) during 2004–2011. Rural communities located within GMAs where sport hunting occurred received on average > 6,000 kgs per GMA of fresh game meat annually from hunting operators. To assess hunting industry compliance, we also compared the amount of meat expected as per the lease agreements versus observed amounts of meat provisioned from three GMAs during 2007–2009. In seven of eight annual comparisons of these GMAs, provisioning of meat exceeded what was required in the lease agreements. Provisioning occurred throughout the hunting season and peaked during the end of the dry season (September–October) coincident with when rural Zambians are most likely to encounter food shortages. We extrapolated our results across all GMAs and estimated 129,771 kgs of fresh game meat provisioned annually by the sport hunting industry to rural communities in Zambia at an approximate value for the meat alone of >US$600,000 exclusive of distribution costs. During the hunting moratorium (2013–2014), this supply of meat has halted, likely adversely affecting rural communities previously reliant on this food source. Proposed alternatives to sport hunting should consider protein provisioning in addition to other benefits (e.g., employment, community pledges, anti-poaching funds) that rural Zambian communities receive from the sport hunting industry.

## Introduction

Sport hunting in Africa represents an important form of revenue-generating tourism [1],[2]. Additional reported benefits include conservation of biodiversity through protection of large tracts of land that might otherwise be converted to agricultural or domestic livestock use [3–7] and selective hunting of potentially dangerous or destructive species that can increase local people’s tolerance for coexistence [8–10]. However, others report that sport hunting contributes little to rural economies or livelihoods [11].

Employment, revenues from hunting fees and licenses, contributions to community capital projects, and funding of anti-poaching scouts are potential benefits that rural communities may receive from sport hunting [12],[13]. Access to game meat has been described as the most tangible benefit to rural communities [14]; however, comparatively little effort has been made to characterize meat provisioning. Notable exceptions are Namibian communal conservancies that report annually on game meat revenues attributable to sport hunting [15], and the comprehensive summary of game meat distributions in Africa compiled by TRAFFIC which considered sport hunting in addition to game ranching and culling [14]. In Zambia, a 1971 report based on household surveys estimated that sport hunting provided 112 tonnes of dressed game meat to rural Zambians countrywide [16] (cited in [12]). We are unaware of more recent studies in Zambia, including studies that quantified provisioning at the GMA scale. In addition, we found no record of previous attempts to examine the patterns or scope of meat distributions.

Food security in developing nations is a primary focus of aide organizations worldwide [17–21]. In Zambia, 48% of the population was undernourished in 2012–2014 [22] and 19% of Zambian households were recently classified as chronically food insecure [20] defined as a continuously inadequate diet caused by the inability to acquire food [23]. Food shortage is defined as an individual receiving less than 2,200 kilocalories per day [18]. Donor aide programs provide supplemental food typically consisting of maize, sorghum, or other cereals. Although high-protein cereals are being developed [24], at present recommended daily protein requirements (50 gm/day)[25] are rarely met in Zambia [22],[26].

As part of their official lease agreements with the Zambia Wildlife Authority (ZAWA), sport hunting operators in Zambia are required to provide to rural communities a proportion of game animals harvested by clients each year. This meat is distributed free of charge to villages that reside within game management areas (GMAs) where sport hunting occurs. To legally obtain protein outside of GMAs, rural Zambians must raise their own livestock, fish, or purchase meat. Livestock husbandry is costly and precluded in some areas due to diseases [27], inadequate grazing, or presence of tsetse flies *Glossina* spp. [28]. To fish legally requires purchasing a permit and access to lakes, rivers, or seasonal ponds. Purchasing meat is expensive: about US$10/kg for beef, US$7/kg for chicken, and US$15/kg for dried fish (i.e., kapenta *Limnothrisa* sp.). Most (64%) Zambians live on less than US$1.00/day [29]. Thus, high expense makes obtaining animal protein cost prohibitive for most rural Zambians.

Obtaining sufficient amounts of protein is a widespread problem faced by rural people in many parts of Africa. Alternatives for meeting the protein requirements are being explored in other countries. In particular, game ranching, communal conservancies [30],[31], and other forms of community-based wildlife conservation programs [32] show promise. However, despite a few earlier attempts, these programs in Zambia are limited in scope, highly localized, and poorly developed [14], meaning that currently most rural Zambians do not have access to meat through these means.

Presently, the only other alternative for rural Zambians to obtain meat is illegally through poaching of wild animals or purchase of poached meat (herein referred to as bushmeat). Global concerns over the prevalence and extent of bushmeat poaching are increasing rapidly [33],[34]. Studies cite the unsustainable nature of the bushmeat trade [34] and the loss of potential income that results from poaching compared to the value of the same animals to photographic tourism or sport hunting [35].

We quantified the annual provisioning of fresh game meat by the sport hunting industry to rural communities in Zambia. Specifically, we characterized the expected and observed amounts of meat provisioned and the timing and spatial extent of meat distributions to rural communities. Finally, we discuss meat provisioning in the context of rural livelihoods, in particular food security and bushmeat poaching, in relation to Zambia’s 2013–2014 hunting moratorium.

## Methods

Zambia Wildlife Authority ranks GMAs as prime, secondary, or understocked based on species occurrence and animal abundance [36]. Prime GMAs include species that are fairly abundant and include highly valued trophies (e.g., African lion *Panthera leo*, leopard *P*. *pardus*, roan antelope *Hippotragus equinus*, and sable antelope *H*. *niger*) and that can accommodate five or more classical safaris and seven or more mini safaris per hunting season. Secondary GMAs include species less abundant but that generally can sustain three to four classical safaris and a minimum of five mini safaris per hunting season. Understocked GMAs contain species assemblages that are intact but with populations low enough that no hunting quota or only a minimum hunting quota is allowed [37]. Quotas are set annually for each GMA based on their ranking, an evaluation of the hunting operators’ performance in the GMA during the previous season, and input from regional ZAWA scouts, ZAWA ecologists and representatives of the rural communities located within the GMAs. We obtained quotas from 2007 to 2009 from ZAWA for each of 30 GMAs in Zambia where sport hunting occurred. These 30 GMAs cover 84,033km^2^ and represent 11% of Zambia’s total land area. We excluded non-edible trophy species (African lion, leopard, spotted hyena *Crocuta crocuta*, Nile crocodile *Crocodylus niloticus*, small carnivores, and primates) from all calculations.

Sport hunting operators in each GMA are mandated by their lease agreements to utilize (i.e., hunt) ≥ 60% of their annual quota and distribute ≥ 50% of all game animals harvested each year to rural communities located in the GMA where the hunting occurred. Thus, 60% of the allocated quota of game species represents the estimated minimum number of edible animals harvested annually by sport hunters in Zambia; half of which represents the minimum amount of game meat that communities may expect to receive in a given year.

We collected data on meat provisioning from three GMAs (1 prime, 1 secondary, 1 understocked). During the study, each GMA was operated by individual hunting companies under a ten-year (2002–2012) lease agreement with ZAWA and the local communities. Zambia’s sport hunting season occurred each year from 1 May to 31 December; however, sport hunting declines during the hottest months (mid-October to early November) and is typically discontinued when the rainy season begins in late November (P. White, personal observation).

We used reported live [38] and dressed weights [25] of game species to estimate harvested biomass. We used body weights for subspecies that occurred in Zambia when available. For species whose dressed weights were not available, we used conversion rates from a closely related species within the same genus. Reported dressed weights excluded offal; however, rural Zambians routinely consume organ meats (e.g., heart, liver, kidneys) in addition to skeletal muscle and bones which are boiled for additional nutrition (P. White, personal observation). Thus, dressed carcass weights represent minimum estimates of the amount of edible meat.

For each of the three GMAs sampled, we calculated the annual amount of meat expected through provisioning during 2007–2009 (data for 2008–2009 only were available for the understocked GMA). We converted the number and species of animals represented by 50% of the utilized quota to kgs using the same dressed weight calculations as were applied to meat distribution logs (below). We also estimated the expected mean annual amount of meat provisioned in all GMAs where sport hunting occurred during 2007–2009. We excluded GMAs not hunted during the previous lease period, specialized GMAs, private game ranches, and conservancies. We used 60% of the allocated quotas at 50% distribution and converted these values to dressed weights to estimate the total kgs of meat expected to be provisioned countrywide.

We estimated the amount of meat provisioned annually during the 2005–2011 hunting seasons for the sampled prime and secondary GMAs and for five seasons during 2004–2011 for the understocked GMA. We obtained this information from meat distribution logs provided to ZAWA by hunting operators. Logs included date, species, portion of carcass distributed (e.g., half, whole), and generally a description of the recipient location (e.g., name of village, clinic, school, game scout post). Each log entry represented one animal or portion of an animal distributed to one location as verified by signature of the receiving party. We calculated dressed weights (kgs) from species and amount of provisioned meat as described previously.

To assess performance of the sport hunting industry in meeting its meat provisioning requirements, we compared annual expected and observed amounts (kgs) of provisioned meat in the three sampled GMAs during 2007–2009. We also summarized the amount (kgs) of meat provisioned and number of distribution events by month and year. When possible, we determined locations of distribution events using Google Earth, survey charts, and 1:250,000 scale topographic maps (Surveyor General, Lusaka, Zambia) and estimated distances from the recipients’ locations to the provisioning hunting camp to determine the spatial extent of meat provisioning.

We used generalized linear mixed-effects models in program R (version 3.0.2; R Development Core Team 2013) [39] to estimate differences in response variables among GMAs and months. Response variables were the amount of meat provisioned and number of distribution events; explanatory variables were GMA and month. We included year as a random effect. We used least-squares difference means tests to compare differences among GMAs and months. We report means with ± 1 standard deviation and set statistical significance at α = 0.05.

## Results

**Fig 1:**
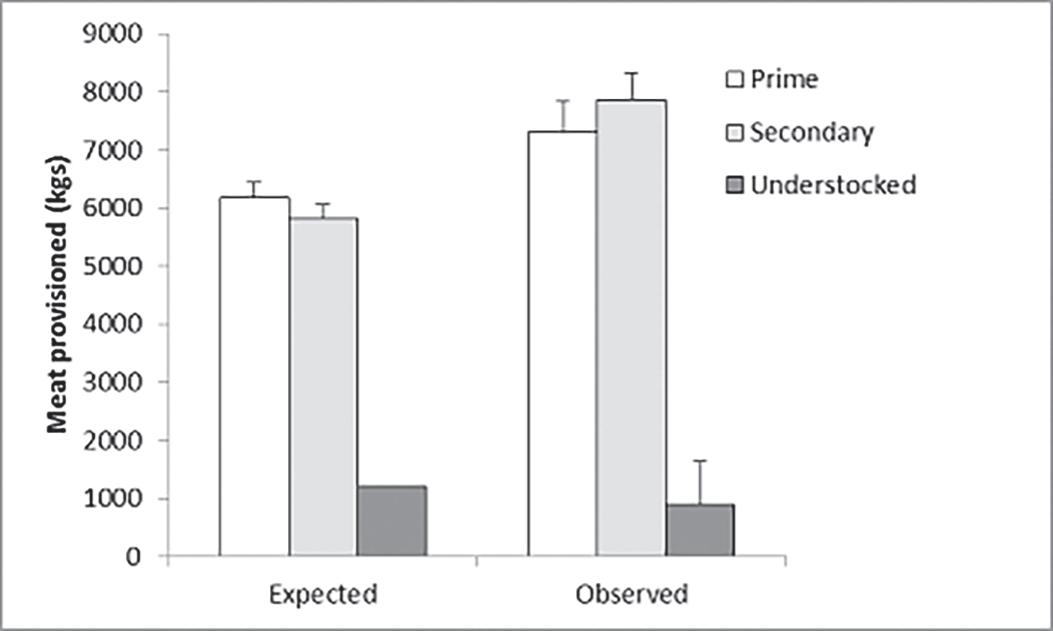
Expected versus observed amounts (kgs) of meat provisioned in each of three Game Management Areas (GMA), Zambia.

Mean expected annual amount (kgs) of meat distributed countrywide was 129,771 ± 15,862 kgs during 2007–2009, representing 2,359 ± 320 individual animals of 20 species (Table 1). The mean expected annual amount of meat provisioned from 2007 to 2009 was greater for the prime (6187.3 ± 262.1 kgs) and secondary (5810.0 ± 248.6 kgs) GMAs than for the understocked (1204.0 ± 0 kgs) GMA. Similarly, the observed amount of meat provisioned annually during 2004–2011 varied among GMAs (*F*_2,16_ = 21.97, *P* < 0.001), with the prime (5832.1 ± 1579.9 kgs; *n* = 7) and secondary (6495.0 ± 1876.1 kgs; *n* = 7) GMAs provisioning greater (*P* < 0.05) amounts of meat than the understocked (973.4 ± 390.3 kgs; *n* = 5) GMA. Considering paired 2007–2009 data only, the observed amounts (kgs) of meat provisioned in prime and secondary GMAs exceeded what was expected each year (Fig. 1). In the understocked GMA, observed amounts exceeded expectations in one of two years.

**Table 1:** Mean (± SD) annual harvest quotas and expected amounts (kgs) of dressed meat provisioned during normal hunting seasons in Zambia, 2007–2009.

	Allocated Quota Countrywide		Mandatory 60% Utilization of Allocated Quota		Mandatory 50% Distribution of Utilized Quota		Expected Dressed Meat Provisioned (kg)	
Species	Mean	SD	Mean	SD	Mean	SD	Mean	SD
Cape buffalo (*Syncerus caffer*)	295.3	20.6	177.2	12.4	88.6	6.2	31,718.8	2217.6
Bushbuck (*Tragelaphus scriptus*)	160.0	24.3	96.0	14.6	48.0	7.3	1344	204.4
Bush pig (*Potamochoerus larvatus*)	56.7	21.1	34.0	12.7	17.0	6.3	629	234.5
Common duiker (*Sylvicapra grimmia*)	95.7	22.3	57.4	13.4	28.7	6.7	315.7	73.6
Eland (*Taurotragus oryx*)	46.3	15.5	27.8	9.3	13.9	4.7	5740.7	1920.8
Lichtenstein’s hartebeest (*Alcelaphus buselaphus*)	89.3	17.2	53.6	10.3	26.8	5.2	2572.8	496.6
Hippopotamus (*Hippopotamus amphibious*)	206.3	23.2	123.8	13.9	61.9	7.0	53853	6067.0
Impala (*Aepyceros melampus*)	344.7	18.9	206.8	11.3	103.4	5.7	3308.8	181.2
Greater kudu (*Tragelaphus strepsiceros*)	117.0	21.4	70.2	12.8	35.1	6.4	4598.1	840.1
Red lechwe (*Kobus leche*)	15.7	4.6	9.4	2.8	4.7	1.4	324.3	95.6
Oribi (*Ourebia ourebi*)	48.3	9.5	29.0	5.7	14.5	2.8	116	22.7
Puku (*Kobus vardoni*)	191.7	10.3	115.0	6.2	57.5	3.1	2357.5	126.2
Southern reedbuck (*Redunca arundinum*)	67.3	11.0	40.4	6.6	20.2	3.3	585.8	95.8
Roan (*Hippotragus equinus*)	45.7	13.6	27.4	8.2	13.7	4.1	2041.3	608.5
Sable (*Hippotragus niger*)	61.3	11.9	36.8	7.2	18.4	3.6	2318.4	451.0
Sitatunga (*Tragelaphus spekei*)	24.3	2.3	14.6	1.4	7.3	0.7	459.9	43.6
Warthog (*Phacochoerus africanus*)	182.0	30.1	109.2	18.1	54.6	9.0	2948.4	487.9
Waterbuck (*Kobus ellipsiprymnus*)	102.0	25.0	61.2	15.0	30.6	7.5	4375.8	1071.6
Wildebeest (*Connochaetes taurinus*)	74.3	18.0	44.6	10.8	22.3	5.4	3122	756.4
Zebra (*Equus quagga*)	135.7	19.6	81.4	11.8	40.7	5.9	7041.1	1017.5
Totals	2359.7	320.6	1415.8	192.4	707.9	96.2	129,771.4	15,861.5

Expected dressed meat provisioned calculated using live and dressed weights of game species after Skinner & Smithers (1990) and Bothma & du Toit (2010), respectively.

**Fig 2:**
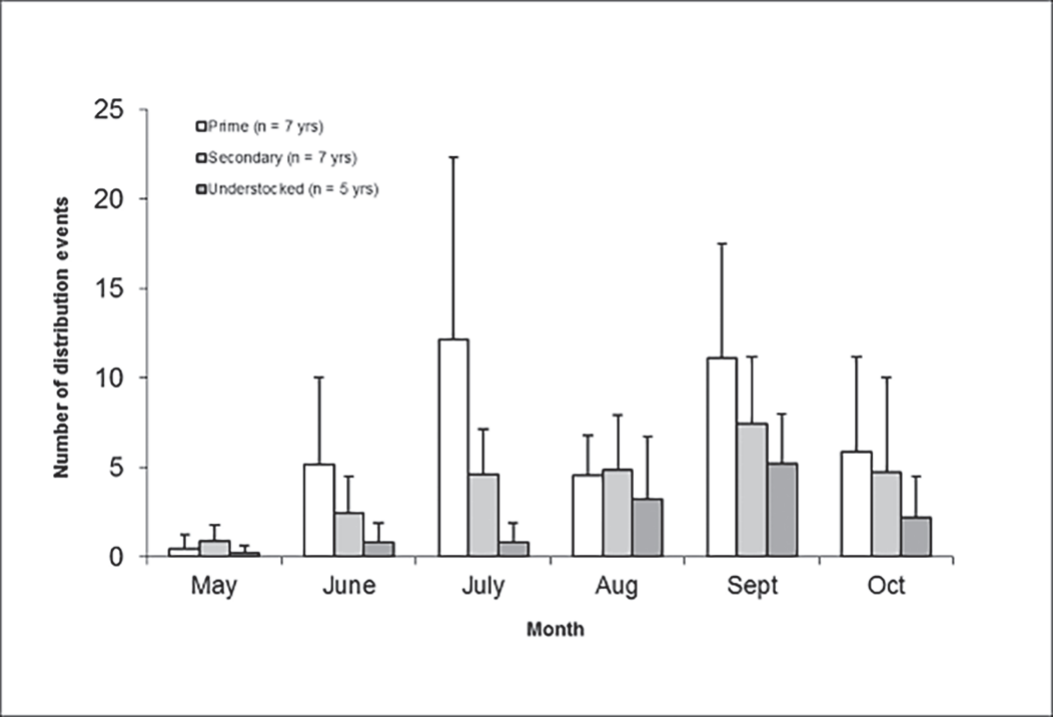
Mean (± SD) number of meat distribution events by month for three Game Management Areas, Zambia.

The mean annual number of distribution events varied among GMAs (*F*_2,16_ = 17.32, *P* < 0.001), with more distribution events (*P* < 0.05) in the prime GMA (39.3 ± 8.7) than in the secondary (24.9± 8.9) and understocked (13.8 ± 8.1) GMAs. Annual number of distribution events in the secondary and understocked GMAs were similar (*P* > 0.05). The timing of distributions varied across months (*F*_5,95_ = 7.13, *P* < 0.001) with more distribution events during July and September and fewer during May (Fig. 2). There was no interaction between GMA and month (*F*_10,95_ = 1.32, *P* = 0.233).

**Fig 3:**
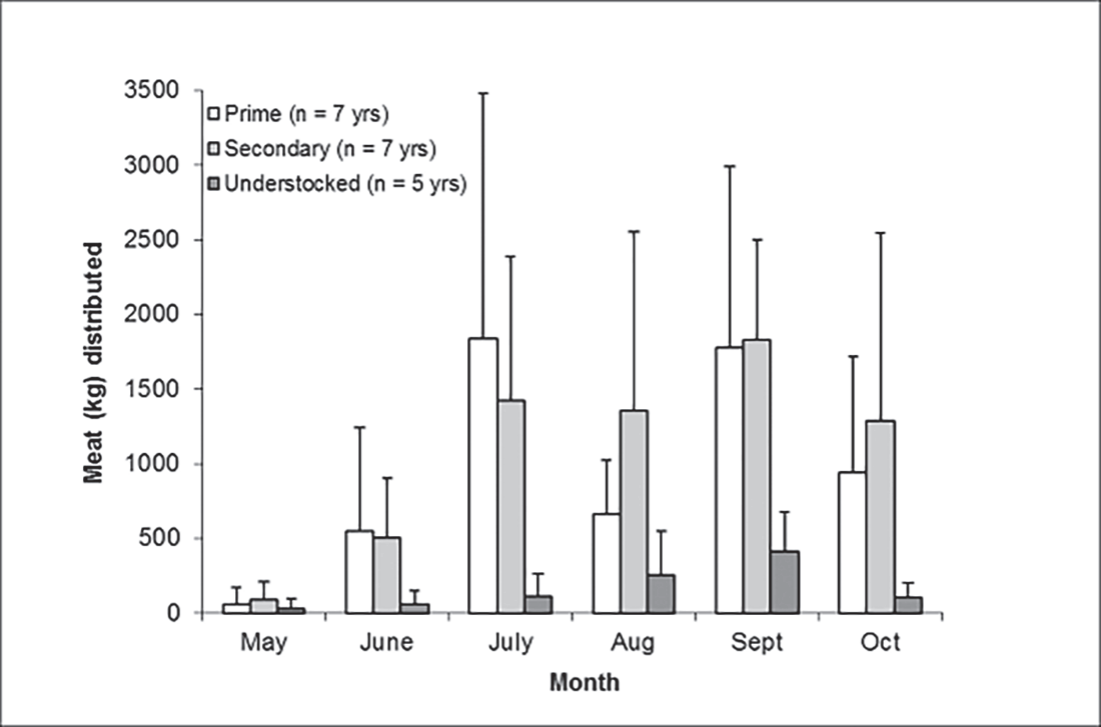
Mean (±SD) amount (kgs) of meat distributed by month for three Game Management Areas, Zambia.

The observed amount (kgs) of meat provisioned each month varied among GMAs (*F*_10,95_ = 1.24, *P* = 0.277) with greater amounts of meat provisioned from the prime and secondary GMAs than from the understocked GMA (*P* < 0.05; Fig. 3). Overall, mean total amounts (kgs) of meat provisioned were greatest in September and least in May (*F*_5,95_ = 6.15, *P* < 0.001). There was no interaction between GMA and month (*F*_10,95_ = 1.32, *P* = 0.233). In the prime GMA, mean meat provisioning in July and September exceeded 1,700 kgs/month. In the secondary GMA, mean monthly provisioning was 1,800 kgs in September and exceeded 1,250 kgs/month during July–October. The greatest mean monthly amount of meat provisioned in the understocked GMA was in September (> 400 kgs).

**Fig 4:**
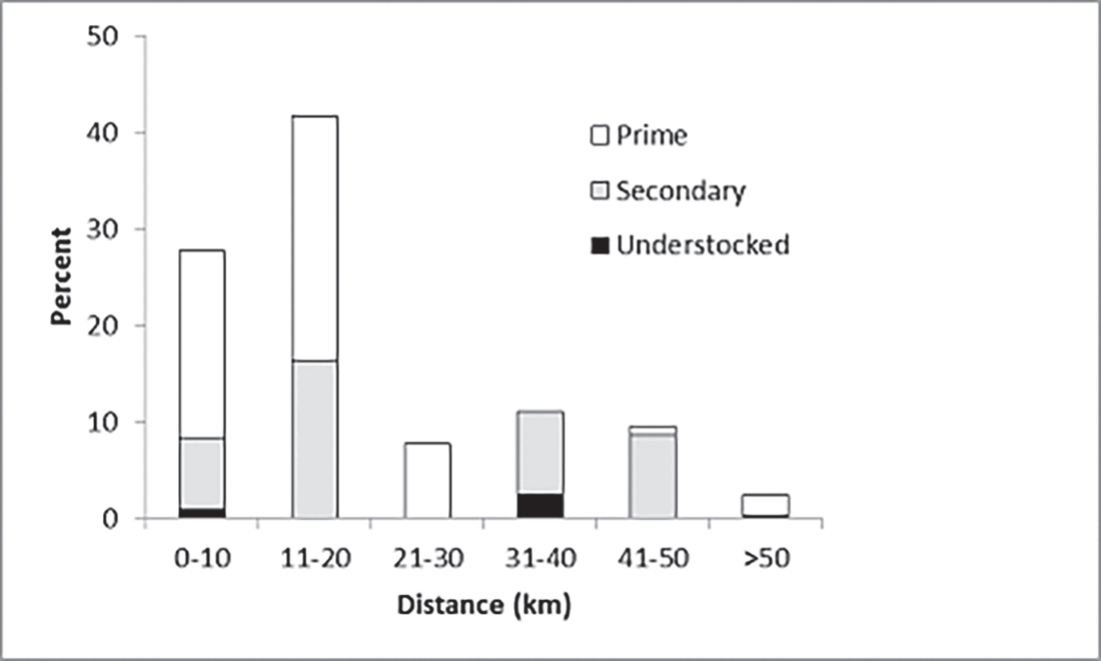
Distances from sport hunting camps of meat provisioned from three Game Management Areas, Zambia.

Distribution logs contained locations of meat recipients in 62% (*n* = 518) of entries, including 63% (*n* = 275) in the prime GMA, 78% (*n* = 174) in the secondary GMA, and 16% (*n* = 69) in the understocked GMA. Remaining entries were received by community-appointed liason officers who oversaw onward distribution. To assess the spatial extent of meat provisioning, we determined specific locations for 336 distribution events in relation to the locations of hunting camps that supplied the meat. Meat provisioning extended to > 50 km from sport hunting camps with most (70%) occurring within 20 km of camps (Fig. 4).

## Discussion

In Zambia, provisioning of fresh game meat by sport hunting operators constituted an important source of protein for rural communities, with actual meat provisioned exceeding 6,000 kgs annually in the sampled prime and secondary GMAs. Meat provisioning by the hunting industry appeared reliable across years and exceeded expectation in each GMA and in every year except one (understocked GMA in 2009). Variations in the amount and timing of distributions appeared influenced in part by unpredictable short-term perturbations. For example, eruption of the Icelandic volcano and subsequent disruption to international flights in 2010 [40] caused the abrupt cancellation of numerous hunts and lowered the amount of meat distributed in some months that year (PHAZ, personal communication).

The provisioning of greater amounts of meat than expected resulted when operators successfully hunted more than the minimum 60% required of their annually allocated quota (P. White, unpublished data). These results illustrate a potential conflict of interest; ZAWA receives revenues based on GMA ranking (prime GMAs carry the highest fees) as well as the number of animal licenses sold. Therefore, ZAWA could be motivated to rank GMAs as high as possible and to allocate larger quotas in order to receive greater revenues from hunting operators [37]. Local communities also receive a portion of each animal license fee, and receive more meat when allocated quotas are high, especially for large-bodied species. Thus, community representatives engaged in quota setting exercises may also be motivated to support high quotas.

Likewise, hunting operators may be motivated to sell as many hunts as possible each year to maximize profits, especially in prime GMAs where fees are greater. However, an operator’s future success is gauged on quality of trophies obtained in previous years. Trophy quality is judged on size and age rather than number of animals shot [41]. Further, lease periods of ≥ 10 years help promote good stewardship because holders of longer-term leases have a vested interest in conserving wildlife populations [42]. Zambian Wildlife Authority requirements of a minimum annual harvest could promote shooting of immature animals if allocated quotas are excessive. Despite existing protocols for post-season meetings designed to set quotas in accordance with best available information, allocated quotas are subject to later change by ZAWA’s head office sometimes without consulting regional ecologists, hunting operators, or local communities. Subsequently, hunting operators occasionally request reduced quotas for certain species if they believe the allocated quota to be excessive in their GMA (PHAZ, personal communication). A more standardized methodololgy for monitoring trends in game population abundance, preferably including estimates of precision, could simplify setting of quotas and help ensure sustainable harvest.

The amount of meat and frequency of meat distributions was generally greater during months (e.g., September) when rural people are most likely to experience food shortages i.e., when alternative sources of food are exhausted and prior to annual crop production [43]. With a hunting moratorium in place since January 2013 throughout most of Zambia’s GMAs, game meat supplied by the sport hunting industry has been almost eliminated. Over 60% of Zambia’s human population was classified as rural in 2013 and 83% of rural Zambian households are poor [44]. Most (20 of 30) of the GMAs analyzed in this study are in three of Zambia’s poorest and most remote provinces (Northwestern, Eastern, and Southern) [44]. Zambia’s poorest families whose crop production is insufficient to support themselves rely on income from casual labor to purchase food during shortages [43]. Food is the largest expenditure for Zambia’s rural poor [45] for whom casual labor constitutes a major source of income [46]. However, most employment opportunities provided by sport hunting camps each year were lost during the current moratorium. This included casual work (road clearing, camp building) as well as seasonal jobs that lasted throughout the hunting season (camp staff, trackers and skinners, drivers and mechanics, etc.). In addition, commodities normally purchased by hunting camps from local communities (e.g., thatching grass, vegetables, maize meal) were not needed (P. White, unpublished data). Thus, in addition to the loss of provisioned meat, many rural people may lack the financial resources to legally purchase meat. During the similar time period (February 2013–February 2014) inflation in Zambia increased 7.6% [47]. The rate of inflation has since increased to 8.1% (November 2013–November 2014) with the cost of food contributing the most to the annual increase [48]. Zambia’s poorest provinces have experienced the highest inflation rates countrywide [48].

In the absence of sport hunting, rural communities need alternatives to reliably and legally obtain meat. Solutions must be appropriate to local situations however [34], and consider the needs of humans and wildlife to avoid adverse effects [49–51]. Domestic livestock are excluded from many parts of Zambia due to tsetse flies [28] and can cause habitat degradation if overstocked [52]. Game ranching can provide a source of meat and African game meat has a greater protein content (> 20% per 100g) than domestic species (< 20% per 100g) [25]. But whereas sport hunting operators provision free meat as part of their lease agreements, ranchers may expect fair or subsidized prices for their product [53]. A shift to greater reliance on wild fisheries can result in depletion of fish stocks [33]; Zambia’s native fish stocks are showing evidence of severe depletion [54],[55]. Communal conservancies that incorporate game and domestic stock could provide rural people with protein and income. These programs require considerable start-up investment and maintenance and a sport hunting component is often needed to ensure success, especially in the early stages [56],[57]. The most profitable conservancies include a sport hunting component [7],[15]; however, the income received by individuals is inversely related to human population density [10]. Benefits to residents diminish when conservation-benefit programs result in an influx of immigrants seeking to share in returns [58],[59].

Where alternative sources of food and income are not available, incidents of bushmeat poaching increase during the late dry season [35],[60],[61]. Many hunters sell bushmeat to obtain cash to purchase other foods [34], material goods [60], or to enhance their farming efforts thereby increasing the income potential of their crops [62]. Whether hunting for food or cash, expanding human populations coupled with the unsustainable hunting methods favored by bushmeat poachers have caused widespread concerns for the viability of wildlife populations and entire species [34],[63],[64]. Studies of bushmeat hunters in Zambia [60] and buyers in Zimbabwe [53] concluded that increasing the supply of free meat would help reduce bushmeat poaching.

Those most dependent on bushmeat for food are often the most remote and marginalized groups [33],[34],[49]. In Zambia, poor families are more likely to live ≥ 20 km from the nearest markets than families that are not poor [45]. Most of Zambia’s GMAs are located in the poorest and most remote provinces [44]. Sport hunting operators in Zambia provisioned meat to at least 50 km from their hunting camps, which were situated in remote areas on average 84 km from the nearest towns (*N* = 32 camps). While the price of bushmeat can vary with distance to source [65], average cost of bushmeat in Zambia in 2012 was US$4.75/kg of fresh game meat (P. White, unpublished data). Thus, the estimated replacement cost of game meat exceeds US$27,700/year for each prime GMA, US$30,850/year for each secondary GMA, and US$4,620/year for each understocked GMA. Countrywide, the estimated annual cost to purchase the equivalent of 129,771 kgs of fresh game meat is US$616,412, excluding butchering and delivery costs. At > 20% protein per 100g game meat, this is equivalent to 519,084 people days of protein per year. This figure is based on the recommended daily protein requirement of 50 gm/day [25] being fulfilled entirely by animal protein. In fact, Zambians on average obtain less than 20% of their dietary energy from animal protein [66]. Thus, although meat provisioned by sport hunting operators represents a small percentage of protein requirements for Zambians, it appears an effective means of distributing fresh, high quality meat to some of the most remote areas of the country with the greatest protein needs, thereby partially alleviating protein deficiencies in rural Africans. Elsewhere in Africa (e.g., Selous Game Reserve, Tanzania) where communities may be located too far from sport hunting camps to facilitate distribution of fresh meat, alternative sources of protein may be required.

The recent moratorium on sport hunting throughout much of Zambia created a crisis situation [67]. Bushmeat poaching is a serious problem in Zambia [14],[37] and the severity and rate of poaching has escalated during the current hunting closure [68]. Similarly, accelerated loss of wildlife occurred during previous (2000–2002) hunting closures in Zambia [4] and elsewhere (Kenya [9],[69]). Zambia’s human population continues to increase (2.8% in 2010) [70]. From 1987–1997, Zambia’s wildlife sector underperformed with regards its potential to supply game meat to rural people [14]. This included game ranching and cropping schemes in addition to sport hunting.

We characterized and quantified meat provisioning in Zambia. Large-scale sport hunting also occurs in the African countries of Namibia, Mozambique, Tanzania and Zimbabwe. In all five countries, over 60% of the total population is rural, with at least 25% of the population in each country categorized as undernourished [22]. With the exception of Namibia, each of these countries has lower than average GDPs compared to the mean for all African countries [71]. Globally, Zambia, Mozambique and Tanzania each rank in the bottom 20 of food supply of meat [66]. As in Zambia, most hunting areas in these other four countries are located in rural regions [72]. National governments and wildlife authorities are responsible for establishing hunting quotas and lease agreements including meat distribution requirements, thus extrapolation of meat provisioning in Zambia to other countries would be speculative, and wider-scale estimates are beyond the scope of this paper. However, the detailed information we provide for Zambia may serve as a template for future investigations of expected meat distributions in other countries or regions where hunting occurs.

While the amount of game meat provisioned annually by Zambia’s sport hunting industry is substantial, a multi-use approach that incorporates sport hunting, game ranching, and communal conservancies for additional sources of protein and income in rural areas will likely be required to keep pace with the growing demand for meat and jobs. It is vital that Zambia’s GMAs be afforded sound custody unless and until viable alternatives offering the same or greater benefits than sport hunting provided can be implemented.
